# Ventricular Tachycardia Catheter Ablation: Retrospective Analysis and Prospective Outlooks—A Comprehensive Review

**DOI:** 10.3390/biomedicines12020266

**Published:** 2024-01-24

**Authors:** Laura Adina Stanciulescu, Radu Vatasescu

**Affiliations:** 1 Cardio-Thoracic Department, “Carol Davila” University of Medicine and Pharmacy, 050474 Bucharest, Romania; radu_vatasescu@yahoo.com; 2 Cardiology Department, Clinical Emergency Hospital, 014461 Bucharest, Romania

**Keywords:** ventricular tachycardia, catheter ablation, structural heart disease, cardiovascular, arrhythmia

## Abstract

Ventricular tachycardia is a potentially life-threatening arrhythmia associated with an overall high morbi-mortality, particularly in patients with structural heart disease. Despite their pivotal role in preventing sudden cardiac death, implantable cardioverter-defibrillators, although a guideline-based class I recommendation, are unable to prevent arrhythmic episodes and significantly alter the quality of life by delivering recurrent therapies. From open-heart surgical ablation to the currently widely used percutaneous approach, catheter ablation is a safe and effective procedure able to target the responsible re-entry myocardial circuit from both the endocardium and the epicardium. There are four main mapping strategies, activation, entrainment, pace, and substrate mapping, each of them with their own advantages and limitations. The contemporary guideline-based recommendations for VT ablation primarily apply to patients experiencing antiarrhythmic drug ineffectiveness or those intolerant to the pharmacological treatment. Although highly effective in most cases of scar-related VTs, the traditional approach may sometimes be insufficient, especially in patients with nonischemic cardiomyopathies, where circuits may be unmappable using the classic techniques. Alternative methods have been proposed, such as stereotactic arrhythmia radioablation or radiotherapy ablation, surgical ablation, needle ablation, transarterial coronary ethanol ablation, and retrograde coronary venous ethanol ablation, with promising results. Further studies are needed in order to prove the overall efficacy of these methods in comparison to standard radiofrequency delivery. Nevertheless, as the field of cardiac electrophysiology continues to evolve, it is important to acknowledge the role of artificial intelligence in both the pre-procedural planning and the intervention itself.

## 1. Introduction

Ventricular tachycardia (VT) is a significant cardiac arrhythmia that is associated with increased mortality, especially in patients with structural heart disease (SHD). Sudden cardiac death (SCD) (6.7 per 100.000 person years) is responsible for roughly 25% of cardio-vascular mortality, with a significant proportion of this produced by malignant ventricular arrhythmias (VAs), i.e., hemodynamically unstable VT or ventricular fibrillation (VF) [1].

Since their introduction, implantable cardioverter-defibrillators (ICDs) have revolutionized the treatment of individuals experiencing malignant VAs, emerging as a class I recommendation for patients with both ischemic and nonischemic cardiomyopathy who are susceptible to SCD [2,3]. Although they have the capacity to successfully terminate VT episodes, they are unable to prevent them, as the arrhythmogenic substrate is not influenced by antitachycardic pacing (ATP) or internal shocks. Moreover, ICD therapies (shocks and, to a lesser degree, ATP as well) have been associated with a higher morbi-mortality rate and a significant deterioration in the overall quality of life (QoL) [4,5,6]. A combined regimen of betablocker therapy and amiodarone demonstrates a potential mitigation of ICD shocks for select patients [7], but it is imperative to acknowledge that amiodarone is accompanied by notable side effects, leading to a cessation of drug usage in nearly a quarter of the patient population.

Catheter ablation has emerged as the preferred treatment option for patients with either ischemic or nonischemic VTs and has persistently proved to be superior to antiarrhythmic drugs (AADs) in decreasing the recurrence rate in patients with SHD, especially in patients with a high risk of recurrence, such as those who are refractory to usual AADs regimens or those with secondary prevention [8,9,10]. Despite catheter ablation becoming a common procedure in highly specialized electrophysiology (EP) centers, accurately identifying the critical isthmus and effectively ablating all inducible VT morphologies may be challenging. Moreover, discerning the optimal approach and technique tailored to each patient’s unique needs can be intricate [11].

In this review, we set out to provide a comprehensive overview on the progress that has been made over the course of the last several years in the ablative treatment of patients with VT, novel therapeutic options for patients with recurrent or refractory VT, as well as the engagement and prospective ramifications of employing artificial intelligence (AI) in the field of electrophysiology.

## 2. Retrospective Analysis and Historical Overview

Understandably, the target population of the first invasive measures aimed at terminating VT circuits were patients with ischemic heart disease, as myocardial infarction (MI) survivors in the pre-reperfusion era were left with large scars which ultimately maintained the re-entry circuits for VT [12].

In 1955, Charles Bailey performed a surgical excision of a myocardial aneurysm for the first time in an ischemic patient with drug refractory VT. Four years later, the patient was still free from VAs, despite interrupting his antiarrhythmic treatment [13,14]. This performance marked the beginning of the surgical VT treatment.

As coronary artery bypass graft (CABG) interventions gained momentum, spectacular results were observed regarding the reduction in arrhythmic burden in patients with previous MI who were surgically revascularized, which prompted cardiovascular teams to adopt concomitant aneurysmectomy and coronary revascularization as the preferred surgical treatment for refractory VT. However, the management of VT was inconsistent and did not justify the risks and high overall mortality of open-heart surgery when the only reason for performing it was the treatment of VT [15,16,17].

In the 1970s, Josephson and his colleagues suspected that the area responsible for initiating and perpetuating the VT circuits was not limited to the scar and extended to the excision beyond the aneurysm for several centimeters to include the healthy tissue surrounding it. The technique was based on pre- and intraprocedural epi-endo activation mapping after VT inducement and was later referred to as the “Pennsylvania peel”. When VT originating from the nearby structures was recorded, such as the papillary muscles or the myocardium beneath the “peeled” subendocardium, adjunctive cryoablation was performed, with relatively good short- and long-term results in terms of arrythmia recurrence. However, this was associated with a high periprocedural mortality (ranging between 5% and 15%), especially in ischemic patients [18,19,20,21,22].

Once the endocardium was established as the prevailing substrate, developing a percutaneous technique for the treatment of ischemic VT was the next step and, naturally, catheter ablation emerged as an alternative approach. The first procedures were described by Hartzler who, in 1983, performed the first VT ablations by delivering 300 J direct-current intracardiac shocks in one patient with VT originating from the right ventricular outflow tract (RVOT) and two patients with previous MI and septal VT [23].

Despite being groundbreaking, the delivery of direct-current intracardiac shocks led to concerns regarding barotrauma with general anesthesia and they were soon replaced by radiofrequency energy, which uses alternative current at 300 to 750 kHz in order to create resistive heating of the target tissue and allows for a more tailored procedure [24,25].

Another milestone was reached when VT re-entry circuits were mapped for the first time using different approaches to assess the 12-lead electrocardiogram (ECG) during the arrhythmia and pace mapping, in order to streamline the activation map and better define the ablation target [26,27,28].

As scar-related VTs are mostly based on an endocardial substrate, further developments in the intracardiac recordings acquisition process and interpretation helped decipher the mechanisms and nature of re-entry circuits. Using activation mapping to detect diastolic activation and entrainment mapping to validate recordings from crucial components of the VT circuit became essential in identifying the critical isthmus site suitable for targeted radiofrequency ablation [12]. In 1993, Stevenson et al. identified the components of the re-entry circuit, while trying to suggest a series of criteria to identify the regions that give rise to re-entry after MI based on the response during entrainment. His description of the circuit, including the exit site, a central isthmus, the entrance site, an inner and an outer loop, as well as bystanders, is still valid today and remains a reference point in circuit-mapping-guided VT ablation [29].

VT circuits, however, are not limited to the subendocardium and, in patients with nonischemic substrate, the circuits are more likely to involve the midmyocardium and/or the epicardium, which is why in these cases there is a high probability of the traditional endocardial approach failing due to its inability to deliver transmural lesions.

In 1996, Eduardo Sosa, facing the Chagas endemic in Brazil and subsequently the high prevalence of epicardial VT associated with the Chagas cardiomyopathy, pioneered percutaneous epicardial access in patients without pericardial effusion to successfully map and ablate their VT circuits [30]. This broadened the scope of catheter mapping and ablation beyond the endocardium to encompass the epicardial surface, which continues to be crucial in situations where the arrhythmic substrate is inaccessible using the classic approach.

Over time, the evolution of electro-anatomical mapping (EAM), enabling a direct visual correlation between anatomical structures and electrophysiological properties, represented a pivotal advancement in the ongoing progress of VT catheter ablation [31].

Marchlinski et al. first described a substrate-based ablation approach for patients with unstable, otherwise unmappable, VTs that did not necessitate precise VT re-entry circuit mapping. They established a cutoff of 1.5 mV to differentiate between normal and abnormal bipolar voltage and performed linear lesions from the scar to the anatomic boundaries or normal endocardium. The novel method allowed for the proper tracing of each deployed lesion, in addition to revealing the substrate. The placement of the linear lesion set was based on the VT QRS complex and the pace mapping used to replicate the VT QRS complex. This, combined with a linear lesion measuring 3 to 4 cm, effectively disrupted VT circuits of substantial anatomical scope, thus laying the foundations of modern substrate ablation techniques [32].

**Figure 1:**
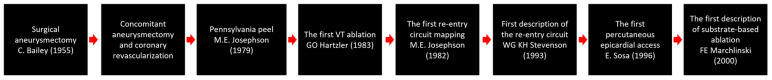
Timeline of technological advancements in the field of VT catheter ablation.

These foundational principles, groundbreaking in their respective eras, persist in contemporary practice, serving as the underpinning for the more advanced techniques currently employed in the field. Their evolution in time is summarized in Figure 1.

## 3. VT Ablation in the Present Time

### 3.1. Ablation Principles and Techniques

Planning for a VT catheter ablation starts with effective strategic planning, which is essential to the seamless execution of the procedure. Beginning the analysis with the examination of 12-lead ECGs, or the VT cycle lengths, and near- and far-field electrograms in patients with ICDs serves as a valuable initial step in discerning between clinical and nonclinical arrhythmias induced during the ablation procedure. Multi-modality imaging enhances pre-procedural planning by depicting scar tissue and uncovering potential arrhythmogenic substrate. This information can be invaluable in precisely delineating the ablation target [33,34].

### 3.2. Types of Catheter Ablation

The main purpose of VT ablation is to target the isthmus components of the re-entrant VT circuit or the origin of automaticity in focal VAs, such as ventricular premature depolarizations (VPDs). There are four mapping techniques that consistently play a role in successful VT ablation: activation mapping, entrainment mapping, pace mapping, and substrate mapping.

### 3.3. Activation Mapping

Activation mapping can be performed in patients with hemodynamically tolerated VTs to assess the myocardial activation and isolate target critical isthmuses or the origin site for focal VTs and VPDs. This entails recording the local electrograms throughout the ventricles and comparing them to the QRS onset during the arrhythmia. The 3D EAMs that are currently available use a color-coded annotation system to record the activation times of different sites in order to identify the localization of the earliest activation. Local electrograms longer than 20 ms before the QRS onset usually indicate the origin site of the arrhythmia and this can be used alongside pace mapping in the ablation of focal VTs.

Conversely, in macro-re-entrant VTs, electrograms recorded before the QRS onset are indicative of the arrhythmia’s exit site, where the circuit meets the first point of myocardial activation. Critical isthmuses in scar-related VTs display low-amplitude diastolic activation and fractioned signals, proving the existence of slow-conducting sites within the dense scar.

Activation mapping has proved to be very helpful, although it has certain limitations when not combined with entrainment mapping, as other components of the re-entry circuit may show mid-diastolic activation despite not being critical and late activation far away from the circuit may show pre-systolic timing despite not being the real exit point of the arrhythmia.

### 3.4. Entrainment Mapping

Entrainment proves valuable in exploring stable, organized re-entry circuits. Typically, the path of re-entry is anatomically consistent and characterized by a fixed and/or functional conduction block and wavefront collision.

Entrainment involves systematically resetting the re-entry circuit by pacing at a constant cycle length faster than the tachycardia itself. This resetting is achieved through the entry of stimulated wavefronts into the circuit, with orthodromic wavefronts traveling through the circuit and colliding with the following paced antidromic wavefront. When the pacing stops, the last orthodromic wavefront finalizes the circuit without any kind of collision and the tachycardia restarts at baseline if the conduction of the circuit has not been affected by the pacing. Following a couple of stimuli, a sustained site of collision is set, with a stable fusion and a stable fused QRS morphology.

During the VT ablation, entrainment maneuvers can be performed to prove the role of a certain area in the re-entrant circuit. The critical elements of the re-entry circuit (entrance site, critical isthmus, and exit point) are those that must be activated for the initiation of the arrhythmia and, therefore, arrhythmia termination can be obtained upon ablation at these sites. In the same fashion, ablation of the other parts of the circuit (inner and outer loops and bystanders) has not proved its role in the termination of the VT despite recordings of mid-diastolic and pre-systolic potentials [35,36].

Upon pacing in the anatomically constrained areas, the paced QRS configuration will be the same as the QRS during the clinically displayed VT and it will show a post-pacing interval (PPI) similar to the cycle length of the arrhythmia and the same local activation order. The exit sites, central isthmuses, and entrance points can then be randomly labeled and further identified, as they show different stimuli to QRS onset interval durations. Ablation at these sites is more likely to bring the tachycardia to an end compared to the noncritical sites.

However, entrainment mapping is limited to hemodynamically stable patients and is not suitable for those with an intramyocardial re-entrant circuit.

### 3.5. Pace Mapping

Pace mapping was developed as a reasonable alternative for focal VT patients in whom activation mapping failed due to the impossibility of arrhythmia inducement or for those with re-entrant circuits in which activation and entrainment mapping was limited due to hemodynamical instability or VT change in configuration upon entrainment.

During pace mapping, the ventricular myocardium is stimulated in order to mimic the morphology of the clinical VT in sinus rhythm. This can be performed at multiple sites during the ablation and has proved to be very effective in identifying critical sites and areas with slow conductive properties.

Ventricular pacing can generate a QRS complex identical to the one during the VT, localize the site of origin and even suggest the potential exit site of the arrhythmia. Despite its low spatial resolution, pace mapping can be very helpful in re-entrant VTs to effectively guide the catheter manipulation by comparing the obtained QRS morphology to the one during the clinically observed arrhythmia [37,38].

### 3.6. Substrate Mapping

Substrate mapping is a reasonable option for patients with noninducible or unmappable VT or those hemodynamically unstable upon arrhythmia induction.

The mapping is performed during sinus and paced rhythm based on the 3D EAM systems that can display voltage maps and, indirectly, define the scarred areas. A voltage >1.5 mV is considered normal, while one <0.5 mV is suggestive of a dense scar. Additionally, the currently available mapping systems (CARTO, NavX, and Rhythmia) can combine voltage maps with activation maps, potentially unveiling critical re-entry components that possess particular properties such as slow or delayed activation.

The CARTO system utilizes magnetic localization technology to determine the position of small sensors embedded in the tips of various diagnostic and ablation catheters through triangulation. The system enables fast anatomic mapping, allows respiratory gating, and creates an accurate, high-resolution chamber geometry reconstruction with overlapped voltage amplitudes, thus becoming a widely chosen option for scar-related VT ablations.

The NavX system’s ability to determine location relies on a 5.6 kHz signal applied alternately across three pairs of skin patches attached in orthogonal planes on the patient’s skin. While NavX has a slightly lower spatial accuracy, both systems can nonfluoroscopically visualize all catheters, paving the road for a completely fluoroless procedure, and they both offer the option of a supervised transseptal puncture, using intracardiac ultrasound.

The Rhythmia platform employs a maneuverable small-basket-array catheter equipped with 64 electrodes to generate a high-resolution EAM, considerably reducing annotation efforts. The catheter is maneuvered within the chamber while displaying electrogram and EAM information. By collecting data from multiple electrodes in each acquired beat, with a significant number of beats per minute, it can effectively create a high-resolution EAM and an ultrahigh-density map with minimal user intervention. There are multiple substrate-based ablation techniques, including linear lesions within the scar, late potentials or local abnormal ventricular activation ablation, scar homogenization and/or dechanneling, and core isolation [39,40,41,42,43,44].

Defining the endpoints for VT ablation is the key to performing a successful procedure and the traditional ones include physiological endpoints that encompass noninducibility, core isolation of crucial elements of the VT substrate and circuit, demonstrated non-excitability or exit block, scar dechanneling, and/or the elimination of late and abnormal electrograms.

Achieving noninducibility through programmed ventricular stimulation (PVS) serves as the traditional procedural endpoint, and despite its limitations in terms of reproducibility, it continues to be considered the gold standard. Newer endpoints, while complementary in terms of noninducibility, seem to have a physiological basis and demonstrate reproducibility with detailed assessment. They are particularly employed for unmappable arrhythmias undergoing substrate mapping and ablation. Non-invasive PVS through using ICDs a couple of days after the ablation might help to further confirm VT noninducibility. However, whether to repeat the ablation in patients with early post-procedural inducible VT requires further investigation.

The rising interest in substrate-based VT ablation techniques highlights the need for new ablation endpoints beyond noninducibility. For linear ablation lesions, failure to capture with high-output pacing along the ablation lines, and changes in the QRS morphology and conduction block across the line upon pacing from each side of the line have been proposed as endpoints, while for the ablation of late electrograms, novel endpoints include the elimination of late potentials, failure to capture with high-output pacing, and changes in late potential activation [45].

Several studies present conflicting findings: whereas some indicate a clear superiority of substrate-based ablation, others report no significant difference between substrate-based and activation/entrainment-guided VT ablation [11,46].

## 4. Efficacy Trials for Catheter Ablation

Several trials investigating the impact of VT ablation on arrhythmia-free survival and overall mortality faced challenges in patient enrollment, leading to premature discontinuation. Despite these issues, five prospective randomized clinical trials have been published, contributing to our current understanding of VT ablation indications.

For example, the SMASH-VT trial in 2007 explored prophylactic substrate-based ablation following secondary-prevention ICD placement in post-MI patients. The study demonstrated significant improvements in the endpoint of survival free from any appropriate ICD therapy in the ablation group compared to the control group [10].

Similarly, the VTACH trial in 2010 investigated prophylactic VT ablation before ICD implantation in patients with ischemic cardiomyopathy. The intervention group exhibited a higher likelihood of being free from recurrent VAs and an extended time before the first recurrence compared to the control group [47].

In 2015, the CALYPSO pilot trial aimed to determine if ablation is superior to AADs in reducing mortality. However, the trial faced challenges in enrollment and was prematurely terminated [48].

The VANISH trial in 2016 compared catheter ablation to AAD escalation in patients with ischemic cardiomyopathy and ICDs with VT despite current AADs. Although mortality did not differ, the composite primary outcome favored catheter ablation [9]

The SMS trial, focusing on substrate-based ablation, randomized patients with ischemic cardiomyopathy to either substrate-based VT ablation with ICD implantation or ICD implantation only. The study found similar event-free survival but a reduction in spontaneous VA episodes in the ablation group [49].

The FACILE-VT study, focused on prospectively evaluating the outcomes of deceleration zone (DZ)-targeted VT ablation during sinus rhythm (SR), showed promising results. Assuming the the VT re-entry circuit can be accurately predicted based on the conduction abnormalities noted in SR, the study team used high-density isochronal late activation maps to identify the arrhythmogenic regions and set the ablation targets. In addition to confirming the high arrhythmogenic potential of the DZs identified in SR, this approach also offers the benefit of reduced RF delivery [50].

Another interesting clinical trial, VOYAGE, was designed to compare cardiac magnetic resonance (CMR)-aided/guided VT ablations with the standard EAM-guided ones in terms of efficacy, efficiency, and safety. With a defined 12-month follow-up period following a one month blanking phase, the study set out to evaluate whether a procedure that is strictly based on imaging could alleviate inter-operator variability and ultimately lead to a more standardized approach [51].

PREVENT-VT introduced the concept of prophylactic VT ablation in patients with post-MI scars, assessing whether a pre-arrhythmic event ablation procedure could decrease the rate of SCD and VT occurrence in patients with previous myocardial ischemia [52].

Following the SURVIVE-VT trial results [8], in more recent years several studies, such as PARTITA and PAUSE-SCD, have extended the inclusion criteria to encompass both ischemic and nonischemic patients in order to assess the optimal timing for VT catheter ablation, proving once again that early VT catheter ablation reduces VT recurrence, cardiovascular hospitalization, and death, as well as ICD therapies [53,54].

Ongoing trials, such as LESS-VT, are exploring the impact of newly developed catheters for VT ablation in patients with SHD (both ischemic and nonischemic) and recurrent VT episodes that are refractory (not effective, not tolerated, not desired) to optimal antiarrhythmic therapy. All the above-mentioned clinical trials are summarized in Table 1.

## 5. Indications for VT Ablation

Several studies have shown the prognostic role of AADs, and especially amiodarone, in patients undergoing VT ablation, proving that amiodarone use in itself is an independent predictor of post-procedural VT recurrence, regardless of the substrate etiology. Even though amiodarone helps achieve intraprocedural noninducibility faster and is considered to be one of the best antiarrhythmic agents for the suppression of VT, it is also associated with a worse post-ablation mortality as well as greater recurrence, the latter attributable to a temporary masking of late potentials and late abnormal ventricular activities, which ultimately leads to less ablation, an overall worse outcome, and a higher risk of mortality. However, there are significant limitations to these studies, namely, that patients on amiodarone usually have a more severe SHD, a faster disease progression, and a more complex substrate [55,56,57].

Despite a significant time difference between their publishing dates that might explain the differences in indications and levels of evidence, the most recent international guidelines on the management of VAs (the latest European Society of Cardiology (ESC) (2022), American Heart Association (AHA)/American College of Cardiology (ACC)/Heart Rhythm Society (HRS) (2017), and Canadian Cardiovascular Society (CCS)/Canadian Heart Rhythm Society (CHRS) (2020) guidelines) significantly narrow the current gaps in the evidence while offering indispensable evidence-based insights to guide daily clinical practice [2,58,59].

Catheter ablation is recommended as the preferred acute therapeutic strategy for recurrent, drug-refractory VAs, particularly in patients with coronary artery disease (CAD), in all three papers. For patients experiencing electrical storms or recurrent VTs, as well as frequent symptomatic drug-refractory VT episodes or poor tolerance to AADs, catheter ablation is a class I recommendation in both the guidelines and the consensus paper.

The ESC guideline emphasizes catheter ablation of premature ventricular contractions (PVCs) if triggered by drug-refractory PVCs.

Specific recommendations vary, with the AHA/ACC/HRS guideline giving a stronger recommendation for AAD therapy (class I vs. IIb for catheter ablation) compared to the novel ESC guideline, which recommends ablation in patients with CAD and recurrent symptomatic VTs on amiodarone (class I) because of its proven superiority to escalation of antiarrhythmic therapy.

Other notable differences include the alternative approaches for the treatment of patients with VT, with AHA/ACC/HRS giving a IIb recommendation for surgical ablation in refractory monomorphic VT, while the ESC only mentions it as a bailout treatment option.

The timing of VT ablation is debated, with ongoing trials exploring its superiority as a first-line therapy. The efficacy of early VT catheter ablation was already assessed in three large clinical trials: SURVIVE-VT, PARTITA, and PAUSE-SCD, with promising results [8,53,54]. The ongoing VANISH2 (Ventricular Tachycardia Ablation versus Escalated Antiarrhythmic Drug Therapy in Ischemic Heart Disease 2) trial will further assess whether catheter ablation is superior to AADs as the treatment of choice for patients with prior MI, ICD, and sustained VT episodes.

The prognostic benefits of VT ablation in SHD are already well documented. The relevance of catheter ablation is increasing, with class IIa recommendations for various scenarios in the ESC guidelines. Differences exist in recommendations for ischemic and nonischemic cardiomyopathy, with stronger recommendations for the former. Previous gaps in evidence regarding the optimal timing for VT catheter ablation have been narrowed by more recent clinical trials (PARTITA and PAUSE-SCD).

The guidelines concur on catheter ablation for specific conditions like arrhythmogenic epicardial right ventricular outflow tract (RVOT) substrate, PVC-induced cardiomyopathy, and idiopathic VF. For HCM patients, for example, the ESC guideline is the only one that highlights the potential benefits of catheter ablation and, while the ESC recommends catheter ablation as the first option for RVOT and fascicular PVCs/VT, the AHA/ACC/HRS guideline gives a class I recommendation for the use of betablockers and non-dihydropyridine calcium-channel blockers and recommends ablation as a second-line therapy after failed drug treatment.

Despite some arguable differences, all three papers highlight the increasing role of catheter ablation in the management of VT episodes, and this is supported by numerous randomized clinical trials that have proved the benefits of this procedure on both the arrhythmic burden and the survival rate. The guideline indications for VT catheter ablation are summarized in Table 2.

Further studies are needed in order to establish the outcome of the novel approaches, as well as the proper timing for the procedure.

However, for the time being, the discerning evaluation of individual risk and benefit, along with a thorough scrutiny of the guidelines discussed, should direct the diagnostic and therapeutic decisions pertaining to VAs [60].

## 6. VT Ablation in Special Populations

### 6.1. Hemodinamically Unstable Patients

VT circuits can sometimes be hard to expose during the procedure in hemodynamically unstable patients if it was not possible to perform the 3D EAM [32,38]. In selected cases, temporary mechanical circulatory support may be considered [61,62].

For unmappable VTs there are other techniques that could be used, such as linear ablation, ablation of late potentials, ablation of local abnormal ventricular activities, scar homogenization, scar dechanneling, ablation of putative isthmus sites defined with pace-mapping during SR, and core isolation of critical substrate elements [63].

1. Linear ablation lesions: This method involves creating contiguous lesions within the heart’s scarred areas to target VT. It is based on replicating surgical experiences with subendocardial resection. Various techniques and variants have been developed, with a focus on pace mapping to identify VT exit sites and the abnormal substrate regions where linear lesions should be applied. Clinical outcomes show varying success rates, with a 75% freedom from recurrent VT in one study and lower VT recurrence rates in others [32,64,65].
2. Ablation of late potentials: This method targets areas with late potentials, which are abnormal electrical signals in the heart. The definition of late potentials can vary between studies, leading to heterogeneity in ablation targets. Clinical outcomes show that eliminating late potentials can reduce VT recurrence rates, but the limitations include varying definitions and the need for comprehensive mapping to ensure all areas are adequately sampled [39,40,66,67].
3. Ablation of local abnormal ventricular activities (LAVAs): LAVAs are abnormal electrical signals within the scarred regions of the heart. This method involves mapping and ablating these LAVAs. Clinical outcomes have shown that modifying or eliminating LAVAs can reduce VT recurrence rates, but challenges include the need for precise mapping techniques and uncertainties in identifying LAVAs [41].
4. Scar homogenization: In this approach, ablation targets all abnormal electrograms within the scar, as defined by specific criteria. Clinical outcomes indicate that this method can be effective in reducing VT recurrence rates, especially when compared to standard substrate ablation techniques. However, the approach requires further study to refine the endpoint for ablation [42].
5. Ablation of channels (scar dechanneling): This technique focuses on identifying and ablating interconnected activation channels within the scar. Studies have shown that this method can lead to noninducibility of VT and low VT recurrence rates. However, challenges include defining the channels accurately and addressing cases with multiple entrance sites or complex three-dimensional activation patterns [68,69,70,71].
6. Ablation of putative isthmus sites defined with pace mapping: This approach involves identifying VT isthmuses by analyzing abrupt transitions in paced QRS morphologies. It can be valuable in targeting critical areas for ablation. Limitations include uncertainties in the optimal number of pace-mapping points and the spatial resolution of 12-lead ECG analysis [38].
7. Core isolation of critical substrate elements: In this approach, a specific critical area within the dense scar, relevant to the patient’s VT, is targeted for ablation. It focuses on achieving electrical isolation within the core of the identified area. Clinical outcomes show promise in reducing VT recurrence rates, and it has a clear ablation endpoint. However, it relies on the presence of preexistent anatomic barriers to anchor ablation lesions, which can be a limitation [44].

Each method has its advantages and limitations, and the choice of the appropriate technique may depend on the individual patient’s condition and the specific characteristics of their VT. Further research and clinical studies are needed to refine these approaches and improve outcomes for patients with VT.

Patients with advanced heart failure (HF) who undergo VT catheter ablation are typically at a higher risk of developing acute hemodynamic decompensation (AHD). The PAINESD risk score was validated for this specific population in order to identify those that might benefit from an optimized hemodynamic approach and periprocedural mechanical hemodynamic support. A high-risk individual is defined as having a score of at least 15 points, with an overall risk of 24% of further developing AHD [72]. An intraaortic balloon pump (IABP), mechanical hemodynamic support (MHS) devices, and extracorporeal membrane oxygenation (ECMO) have previously been used, although there is no data regarding the best strategy. Even though MHS devices showed better outcomes in acute settings compared to IABP, some of them may be affected by electromagnetic interferences (Impella 2.5), while others lead to a more difficult transseptal approach (Tandem Heart). However, neither of them provides right ventricular support, nor respiratory assistance.

Although highly complex, ECMO is a safe strategy, as it offers an extended periprocedural circulatory support, allowing lengthy ablations to be performed with an improved safety [73].

### 6.2. Nonischemic Cardiomyopathies

Patients without CAD often present with a large variety of VT-generating circuits, which can often involve intramural myocardium or epicardium [35]. The final clinical outcome is significantly different than in patients with scar-related VTs, as a better overall post-procedural evolution was noted in patients with CAD compared to those with nonischemic heart disease [74,75,76].

In some cases, with intramural VT etiology, the arrhythmogenic substrate can be extremely hard to approach using the traditional technique, which is why novel methods are currently being studied as alternatives for this population (needle ablation, transcoronary ethanol ablation, etc.) [77].

The pathophysiological substrate in nonischemic cardiomyopathies (NICMs) is more heterogeneous than in patients with CAD and scar-related VTs, as it presents a large variety of particularities depending on the underlying medical condition. In arrhythmogenic cardiomyopathy (ACM), fibro-fatty deposits are visible within the otherwise healthy myocardium, and they are usually displaced around the tricuspid and pulmonary valve annuluses, and the right ventricular (RV) free wall, while in other etiologies, the nonischemic scar is mostly located around the mitral annulus [78,79]. Patients with hypertrophic cardiomyopathy (HCM) often have a unique form of apical scarring [80], while those with type I Brugada syndrome might have targetable substrate within the epicardial RVOT [81]. Other diseases with epicardial substrate particularities include cardiac sarcoidosis, nonischemic dilated cardiomyopathy, patients with myocarditis sequelae, and those with Chagas disease [82,83,84,85,86].

Despite different baseline characteristics and progression patterns, catheter ablation with substrate-based mapping, alongside pace mapping and late potentials identification is needed, although with an overall lower success rate than in ischemic patients.

A recent meta-analysis compared post-ablation outcomes in both ICM and NICM VT patients, concluding that those with NICM had a higher rate of procedural failure and VT recurrence, despite being generally younger than the ischemic cohort (55.3 years compared to 67.0 years). However, peri-procedural and long-term mortality, as well as the complications rate, were similar between the two study populations [87].

### 6.3. VT Arising from the Purkinje System

Jan Evangelista Purkyně was a pioneering Czech physiologist who, in 1845, described viscous fibers within the ventricular endocardium. Ultimately, the term “Purkinje fibers” was assigned to describe these structures [88].

Idiopathic VTs usually originate from triggered re-entry mechanisms in a pathological Purkinje system but could also involve the left or right outflow tracts, peri-annular pathways, and papillary muscles. Quite often they present as a wide QRS complex tachycardia with a bundle-branch block pattern, they are sensitive to verapamil and usually occur in the absence of SHD, as reported upon multimodality imaging assessment (although they were also noted in patients with SHD) [3,89,90].

During the arrhythmia, two different potentials were recorded at a midseptal level—a diastolic P1 potential and a pre-systolic P2 (which was confirmed as the potential of the left posterior fascicle) [91].

The vast majority of idiopathic VTs are cured by catheter ablation, with a very low rate of peri-procedural complications [3]. Recording P1 potentials during the arrhythmia is essential during catheter ablation, but it can sometimes be difficult to achieve. One limitation could be the method’s reduced signal recording, which could potentially be mitigated by using a multipolar linear catheter with small electrodes, while making sure that the catheter is parallel and in contact with the LV septum [90].

In cases with focal, isolated sources, the ablation target is the point of the earliest activation upon VT inducibility, while in left fascicular VTs the aim is to focus on the afflicted Purkinje fibers with diastolic activity during VT [92,93].

Regardless of the actual mechanism involved, it is essential to acknowledge the role of the ventricular endocardium and specifically the Purkinje system, in the initiation and perpetuation of VAs.

## 7. Alternative Approaches to Radiofrequency Catheter Ablation

### 7.1. Stereotactic Arrhythmia Radioablation or Radiotherapy Ablation

Stereotactic arrhythmia radioablation (STAR) has recently been presented as a reasonable bailout option for therapy-refractory VT and has even been mentioned as such by the 2022 ESC guideline on the management of patients with VAs [2]. STAR administers accurate, concentrated radiation to specific areas within the body while minimizing harm to surrounding healthy tissue and it was previously used to treat a large variety of tumors regardless of their underlying pathological substrate [94]. Its main purpose is an overall reduction in arrhythmic burden by integrating 3D EAM, myocardial scar imaging (LGE CMR, MDCT, and PET-CT), and local, non-invasive dispensing of appropriate radiation doses.

Firstly, the identification of the VT substrate is performed using 3D EAM. The left ventricular geometrical characteristics are provided by various imaging techniques, which are also used for segmentation. The two cardiac meshes are then integrated into a unique model, where the physician contours the structure corresponding to the target substrate. The radioablation is then performed based on a previously developed plan including optimal treatment doses and distribution [95].

It was first introduced in 2012 [96] as a treatment for patients with frequent VT recurrences after catheter ablation and appropriate antiarrhythmic treatment and showed promising short-term results [96,97,98,99,100,101,102,103,104], although further recurrences proved to be quite frequent [105,106,107].

Recently, the first STAR long-term follow-up results were published by Herrera et al., showing a high recurrence rate, despite a significant decrease in overall VT burden (even in patients with at least one previous electrical storm) and a positive safety profile (demonstrated, among others, by the fact that none of the patients developed a high-degree atrio-ventricular block (AVB) upon follow-up, even though the interventricular septum (IVS) was the main treatment target in 75% of the enrolled patients). What is interesting to note is that the VTs observed during the follow-up did not have their source within the intended targeted volume but rather in its vicinity. Sustained VT recurrence in this cohort of 20 patients was noted in 90%, while 60% required a repeat procedure. It is also worth mentioning that patients referred to cardiac radioablation usually present with a severe SHD, a significant substrate, and already have undergone other treatment options (such as optimal AAD therapy and catheter ablation), which could explain the high recurrence rate. However, more studies on larger cohorts with long-term follow-up periods are needed in order to confirm these findings and improve the overall strategy [108].

A promising ongoing clinical trial in this field is RADIATE-VT—the first international, multi-center, randomized controlled clinical trial that aims to assess the safety and efficacy of cardiac radioablation in comparison to the traditional percutaneous catheter ablation in high-risk patients that present with refractory VT. The trial uses a non-invasive radiation-delivering device that can precisely target the pre-segmented arrhythmic substrate and deliver focused radiation beams to that specific area. Short-term outcomes are expected with keen interest upon completion of the enrollment period [109].

However, cardiac radioablation can potentially cause severe cardiac and pulmonary long-term effects. After showing promising results in swine models, the use of particle radiotherapy using protons and carbon ions has already been studied in humans, proving that it can deliver a high local dose to the target myocardium with minimal off-target exposure. Concerns were raised regarding the impact on cardiac implantable electronic devices (CIEDs), as neutron exposure may potentially cause device failure. Larger, multicenter studies are needed in order to establish the feasibility and safety profile of using proton-based radiotherapy for the treatment of VT [110,111].

### 7.2. Surgical Ablation

Despite great achievements in the field of percutaneous catheter ablation for VT in recent years, some circuits, especially those that are not scar-related, cannot always be targeted by using standard methods. For patients with inaccessible circuits, contraindications to transcutaneous epicardial access, or simply with previous procedures where catheter ablation failed, surgical ablation might represent an alternative.

A study by Anter et al. evaluated eight patients with refractory sustained VT (SVT) despite optimal AAD treatment, who underwent catheter ablation (either endocardial or epicardial) that proved to be unsuccessful. Using the 3D EAM acquired during the percutaneous procedure, surgical cryoablation was performed on the previously marked sites. A significant reduction in VT burden was noted in most patients during a mean follow-up period of 23 ± 6 months, as shown by the ICDs in terms of appropriately delivered internal shocks upon interrogation [112].

Another study, by Kunkel et al., also enrolled eight consecutive patients, but this time with refractory electrical storm and contraindications to transcutaneous epicardial access, to undergo surgical ablation using intraoperative electro-anatomical landmarks. Clinical VT noninducibility was noted for all patients and the overall VT burden was reduced during a mean follow-up period of 3.4 ± 1.7 years, as 75% of them remained storm-free [113].

Other populations could benefit from surgical VT ablation, especially those with a more difficult anatomy due to congenital heart disease (CHD), such as patients with Fallot tetralogy, where refractory VT represents a common complication, and who may also require another cardiac surgical procedure for a concomitant medical condition. Caldaroni et al. assessed a cohort of 20 Fallot patients with positive EP studies for VT inducibility, who underwent both surgical pulmonary valve replacement and surgical ablation for VT. Upon a median follow-up period of 6.5 years, VT was noninducible in 84.3% of patients, while the remaining patients received an ICD after the post-operative EPS study. No late mortality and no surgery-related complications were reported at 1 year and 5 years. The rates of VA absence were 94% and 89.5%, respectively [114].

Despite not being the gold-standard procedure, surgical VT ablation with the integration of 3D EAM remains a reliable alternative for patients with either recurrent, treatment-refractory VT, or those with contraindications to classic approaches or indications for other open-heart surgical interventions.

### 7.3. Needle Ablation

In some patients, the VT-responsible substrate may be localized on the epicardial surface, deep within the myocardium or might simply not be visible at all with either multimodality imaging or 3D EAM. Despite several optimizations of the standard approach, endocardial RF lesions may sometimes not be large or deep enough to interrupt the arrhythmic circuit. If an epicardial substrate is suspected, then epicardial mapping and ablation could be performed, provided that the operator is aware of the risk of major complications (including perforation and cardiac tamponade) and has the required skills and experience. However, since there are a series of limitations to this approach (distribution of epicardial fat, vulnerable structures in its vicinity—coronary arteries and the left phrenic nerve, significant substrate deep within the myocardium that is impossible to approach, etc.), alternative options to the classic technique have been developed.

Intramyocardial needle ablation is a technique that has been researched for the past 20 years and mainly involves injecting a small amount of saline solution through a retractable needle electrode into the myocardium, thus creating a space that allows for deeper and bigger lesions and higher-energy delivery at the substrate level.

Multiple studies, both preclinical and clinical, evaluated this technique’s potential as a back-up treatment option for patients with recurrent or refractory VTs with at least one previous failed catheter ablation, with promising results [115,116,117,118,119].

With its ability to reach practically almost every potential ventricular site and perform deeper and larger ablation lesions, while maintaining a relatively acceptable safety profile (which might improve in time, as multiple centers worldwide gain more experience in this field), needle ablation is a strong candidate among the ongoing advanced alternative methods for VT disruption in cases where radiofrequency catheter ablation has failed [120].

### 7.4. Transarterial Coronary Ethanol Ablation (TCEA) and Retrograde Coronary Venous Ethanol Ablation (RCVEA)

TCEA and RCVEA are alternative techniques developed for patients with deep myocardial substrate that cannot be reached through traditional approaches, while also trying to protect the adjacent structures.

TCEA was first described more than three decades ago in canine models [121]. It was further clinically tested by Brugada et al. on three patients with incessant ischemic VTs where transcoronary chemoablation in an arrhythmogenic artery ultimately stopped the VT episodes [122,123] and other reports followed shortly [124,125,126].

The procedure involves the presence and correct identification of the artery that provides the blood supply to the arrhythmogenic tissue and the possibility to selectively cannulate it with an angioplasty balloon. Noninducibility rates upon follow-up ranged from 56% to 84%, although it is currently considered a rather inconsistent alternative to the classic approach [127,128,129].

On the other hand, RCVEA poses a series of advantages, the ethanol dilution degree is higher given the retrograde flow, the risk of additional myocardial injury is overall lower, and it does not involve arterial cannulation; therefore, it is not associated with the potential complications that may occur, and it requires far less technical expertise and equipment than TCEA [130].

After performing an extensive 3D EAM, the VT’s earliest endocardial activation point is detected, and the coronary sinus (CS) is cannulated. The earliest site is then determined in both the great cardiac vein (GCV) and the anterior interventricular vein (AIV) and venograms are performed in order to identify the branches closest to that site. A guidewire is then introduced through the vein, which allows further support for the preloaded angioplasty balloon that follows. Once adequacy is checked, the wire is retracted, the balloon is deployed, and contrast is injected in order to assess the degree of myocardial staining (which is an indirect marker that the target site has been successfully reached). The protocol provides for progressive administration of gradually increasing doses of 96% to 98% ethanol until a therapeutic reaction is noted. Ethanol tissue ablation is eventually indicated by the presence of heightened echogenicity in intracardiac echocardiograms and myocardial staining.

This procedure could present some challenges in patients with an atypical venous anatomy, but there are a couple of available techniques that might be able to mitigate these setbacks, such as the “double-balloon technique”, which can either target remote or extensive areas by blocking collateral blood flow using a second balloon, thus allowing for a proper and effective ethanol delivery [131,132].

### 7.5. Remote Magnetic Navigation

The increased radiation exposure associated with VT catheter ablations led to a shift towards non-fluoroscopic techniques. Remote magnetic navigation (RMN) involves the use of externally controlled magnets that are adjusted to create a magnetic field that guides the positioning of the catheters.

The method allows for enhanced catheter control, reduced radiation exposure, and a theoretically lower risk of cardiac puncture and tamponade due to a softer, more flexible catheter tip. Additionally, the improved catheter tip contact is associated with a significantly larger and more homogeneous lesion.

Several studies have assessed the efficacy and safety of this technique, showing a shorter fluoroscopy and ablation time, as well as a lower VT recurrence rate [133,134,135,136,137].

Additionally, RMN has proved to be particularly useful in patients with VAs and CHD, where structural abnormalities (either primary or secondary to surgical procedures) severely limit the access to the target sites.

A meta-analysis by Blandino et al. which compared RMN-guided versus manually guided VT ablation in terms of long-term outcomes showed a reduced radiation exposure and a better safety profile regardless of the etiology of the substrate, as well as a higher acute success rate in patients without SHD [138]. On the other hand, a retrospective analysis performed by Qian et al. outlines better long-term outcomes and a lower rate of VT recurrence in patients with ischemic heart disease treated with RMN, as well as an overall higher procedure duration early on the learning curve [139,140].

MAGNETIC-VT, a recently finalized multicenter clinical trial, set out to compare manually guided to RMN-guided VT ablation in patients with ICM and ICDs. While the results of the study are pending publication, anticipation exists that they will elucidate and clarify variations in outcomes observed in prior studies.

## 8. Future Perspectives

Despite notable advancements in recent years within the field of VT treatment, the management of intricate arrhythmogenic mechanisms underlying the condition can still pose persistent challenges. Over the preceding decades, the prevalence of AI in clinical practice has steadily increased, with cardiovascular medicine emerging as a progressive domain where the efficacy of AI continues to be substantiated. While we can agree that we do not yet have completely autonomous AI models, we can currently access a large variety of technological innovations that show promising results in terms of diagnostic and procedural efficiency, as well as long-term outcomes.

Catheter ablation planning and performance involves a substantial influx of information, encompassing patient history, prior non-invasive and invasive recordings and multimodality imaging clips, real-time navigation, electrograms, ablation parameters and impact, and subsequent clinical follow-up. The interobserver variability regarding the interpretation of individual electrograms cannot be overlooked and can rarely, if ever, be mitigated. The monoparametric linear models that are currently used prove challenging in effectively characterizing optimal targets, while multiparametric nonlinear models, specifically within the domain of machine learning and deep learning, demonstrate exceptional efficacy in identifying targets of clinical relevance and guiding patient treatment.

Machine learning (ML) has been used to detect, localize, and map VT circuits, showing very good sensitivity and specificity and promising results [141,142].

Perhaps the most fascinating technological development is represented by the digital twin—a generic name given to virtual, AI-based heart meshes that use a large variety of clinical (invasive and non-invasive) investigations to create a personalized model that might be able to assist physicians in their everyday clinical practice. A digital twin is essentially a simulated representation of a tangible system, replicating its actions and reactions to different inputs, which enables anticipatory analysis and enhancement.

Multiple studies have evaluated the use of digital twins to either predict VT exit sites, critical isthmuses localization and morphologies, or to guide catheter ablation, with optimistic results [143,144,145,146,147,148], but clinical real-life validation on human subjects and, ideally, larger cohorts is still awaited.

While notable progress has been achieved, it is imperative to address the prevailing ethical and data-related concerns. AI has the potential to facilitate, expedite, and enhance pre-procedural planning, the ablation itself and the follow-up of patients with VT; however, the implementation ultimately relies on human agency and expertise. Nevertheless, it is undeniable that the integration of digital twins into routine cardiovascular practices, both clinical and invasive, is poised to become a commonplace in the foreseeable future.

## 9. Conclusions

In this comprehensive review, we have summarized the evolutionary trajectory of VT catheter ablation procedures, spanning from the initial historical surgical ablation to contemporary state-of-the-art gold-standard approaches and alternative modalities. Alongside elucidating the established principles governing guided mapping and VT ablation, we have provided insights into landmark clinical trials and the latest guideline-driven indications. The narrative encompasses persistent challenges and future perspectives in this domain, underscoring the pivotal role of current technologies, notably the integration of AI, in both pre-procedural planning and the intervention itself. Given the rapid advancement in cardiac electrophysiology, we anticipate a growing body of scientifically validated evidence supporting the benefits of ablation, positioning it unequivocally as the primary therapeutic choice for VT in the foreseeable future.

## Figures and Tables

**Table 1:** Efficacy trials for VT catheter ablation (in order of appearance in the text).

Reference	Study Population	Number of Enrolled Patients	Procedure Approach	Primary Endpoint	Follow-up	Clinical Outcome
SMASH-VT (Reddy et al., 2007) [10]	ICM ablation with ICD versus ICD alone	128	Substrate-based with sinus rhythm mapping	Survival free from ICD therapies	22.5 ± 5.5 months (mean)	Reduced incidence of ICD therapies in the ablation group
VTACH (Kuck et al., 2010) [47]	ICM ablation with ICD versus ICD alone	110	Pace mapping ± entrainment mapping ± substrate modification techniques	Time to first recurrence of VT/VF	22.5 months (SD: 9.0)	Longer time to recurrence of VT/VF in the ablation group
CALYPSO (Al-Khatib et al., 2015) [48]	ICM with ICD and ≥1 appropriate shock ≥3 antitachycardia pacing therapies for VT: antiarrhythmic medications versus catheter ablation	27	At the discretion of the physician	Feasibility	6 months	Prematurely terminated due to several challenges upon enrollment
VANISH (Sapp et al. 2016) [9]	ICM (patients with ICM and ICDs who have VT randomized to ablation or escalation drug therapy)	259	Activation mapping (all inducible VTs were targeted for ablation; if the induced VA was unstable, substrate mapping was used instead)	Composite of death, VT storm, or appropriate ICD shock	27.9 ± 17.1 months (mean)	Lower rate of the composite endpoint in the ablation group
SMS (Kuck et al., 2017) [49]	ICM with LVEF < 40% (ablation with ICD versus ICD alone)	111	Pace mapping ± entrainment mapping ± substrate modification techniques	Time to first recurrence of VT/VF	2.3 ± 1.1 years (mean)	No difference in time between the first VT/VF recurrence between the two study groups
FACILE-VT (Aziz et al., 2019) [50]	Patients with scar-related VTs (ICM and NICM)	120	Assessing the feasibility of targeted DZ ablation	VT recurrence and mortality	12 ± 10 months (mean)	High correlation between DZ during SR and critical sites for VT
VOYAGE (Lilli et al., 2022) [51]	Compared CMR-aided/guided VT ablations with the standard EAM-guided ones	Still enrolling	Pace mapping ± entrainment mapping ± substrate modification techniques	VT recurrence	12 months	Ongoing
PREVENT-VT (Falasconi et al., 2022) [52]	Chronic post-MI patients with CMR-derived arrhythmogenic scar characteristics randomized 1:1 to either catheter ablation or OMT	Still enrolling	CMR-guided subtrate ablation	Composite outcome of SCD or sustained monomorphic VT, either treated by an ICD or documented with ICM	3 years	Ongoing
SURVIVE-VT (Arenal et al., 2022) [8]	Catheter ablation versus AAD as first-line therapy in ICM patients with appropriate ICD shock for VTs	144	Endocardial substrate-based ablation	Composite of cardiovascular death, appropriate ICD shock, unplanned hospitalization for worsening heart failure, or severe treatment-related complications	24 months	Reduced composite endpoint in the ablation group
PARTITA (Della Bella et al., 2022) [53]	Ischemic and nonischemic DCM with primary/secondary-prevention ICDs (phase A) assigned after the first appropriate shock to either ablation or medical therapy (phase B)	517 initially enrolled (47 in phase B)	Late potentials ablation (or early potentials if there were no late potentials)	Composite of death from any cause or hospitalization for worsening heart failure		Reduced composite endpoint in the ablation group
PAUSE-SCD (Tung et al., 2022) [54]	Patients with cardiomyopathy and monomorphic VT with an indication for ICD implantation (ablation + ICD versus standard medical therapy + ICD)	180	High-density mapping (±epicardial mapping) targeting late and abnormal electrograms within the scar ± isochronal mapping ± scar homogenization	Composite end point of VT recurrence, cardiovascular hospitalization, or death	31 months (mean)	Significant reduction in ICD shocks and ATP therapies. No difference in cardiovascular hospitalization or mortality
LESS-VT (2018)	Safety and effectiveness of ventricular ablation therapy using a new catheter in patients with drug-refractory SMVT in whom VT recurs despite AAD therapy or when AADs are not tolerated or desired	Still enrolling	-	Primary safety endpoint—composite of CV-related and procedure-related major complications through 7 days post index ablation procedure Primary effectiveness endpoint—freedom from SMVT at 6 months and a new or increased dose class I or III AAD at 6 months	-	Ongoing

Abbreviations: VT—ventricular tachycardia; ICM—ischemic cardiomyopathy; ICD—implantable cardioverter defibrillator; VF—ventricular fibrillation; LVEF—left ventricular ejection fraction; NICM—nonischemic cardiomyopathy; DZ—deceleration zone; SR—sinus rhythm; CMR—cardiac magnetic resonance; EAM—electro-anatomic map; MI—myocardial infarction; OMT—optimal medical treatment; SCD—sudden cardiac death; AAD—antiarrhythmic drug; DCM—dilated cardiomyopathy; ATP—antitachycardia pacing; SMVT—sustained monomorphic ventricular tachycardia; CV—cardiovascular.

**Table 2:** Indications for VT catheter ablation as formulated by the latest European Society of Cardiology (ESC) (2022), American Heart Association (AHA)/American College of Cardiology (ACC)/Heart Rhythm Society (HRS) (2017), and Canadian Cardiovascular Society (CCS)/Canadian Heart Rhythm Society (CHRS) (2020) guidelines and their levels of recommendation.

ESC [2]	LoR	AHA/ACC/HRS [58]	LoR	CCS/CHRS [59]	LoR
Incessant VT or electrical storm due to SMVT refractory to AAD.	**I**	In patients with prior MI and recurrent episodes of symptomatic sustained VT, or who present with VT storm and have failed or are intolerant of amiodarone or other AADs, catheter ablation is recommended.	**I**	Patients with first or recurrent sustained VT/VF on maximally tolerated betablocker dose and with an ICD/optimized ICD programming with at least one of the following: Significant symptoms Hemodynamically unstable Significant anxiety Multiple ICD shocks High burden of VT/VF Electrical storm with MVT and **ischemic** cardiomyopathy should undergo catheter ablation.	**First line**
Recurrent episodes of PVT/VF triggered by a similar PVC, non-responsive to medical treatment or coronary revascularization.	**IIa**	In patients with ischemic heart disease and ICD shocks for SMVT or symptomatic SMVT that is recurrent, or hemodynamically tolerated, catheter ablation as first-line therapy may be considered to reduce recurrent VA.	**IIb**	Patients with first or recurrent sustained VT/VF on maximally tolerated betablocker dose and with an ICD/optimized ICD programming with at least one of the following: Significant symptoms Hemodynamically unstable Significant anxiety Multiple ICD shocks High burden of VT/VF Electrical storm with MVT and **non-ischemic** cardiomyopathy should undergo catheter ablation.	**Second line**
Recurrent episodes of PVT/VF triggered by a similar PVC, non-responsive to medical treatment or coronary revascularization in the subacute phase of myocardial infarction.	**IIa**	In patients with NICM and recurrent sustained monomorphic VT who fail or are intolerant of antiarrhythmic medications, catheter ablation can be useful for reducing recurrent VT and ICD shocks.	**IIa**	Electrical storm patients with monomorphic VT in whom AAD therapy was unsuccessful.	**Second line**
Patients with SMVT or SPVT/VF triggered by a PVC with similar morphology and an indication for ICD when an ICD is not available, contraindicated for concurrent medical reasons, or declined.	**IIb**	In patients with ARVC and recurrent symptomatic sustained VT in whom a beta blocker is ineffective or not tolerated, catheter ablation with availability of a combined endocardial/epicardial approach can be beneficial.	**IIa**	Catheter ablation is recommended in patients with monomorphic VT and **ischemic** cardiomyopathy (previous MI) in whom treatment with sotalol or amiodarone has been ineffective.	**Second line (may be considered for first line)**
Patients with CCAD and recurrent symptomatic SMVT, or ICD shocks for SMVT despite chronic amiodarone therapy, in preference to escalating AAD therapy.	**I**	In patients with Brugada syndrome experiencing recurrent ICD shocks for polymorphic VT, intensification of therapy with quinidine or catheter ablation is recommended.	**I**	Epicardial mapping should be considered in patients in whom previous endocardial catheter ablation has failed and an epicardial substrate is suspected.	**Second line**
Patients with CCAD and recurrent symptomatic SMVT, or ICD shocks for SMVT despite betablockers or sotalol treatment.	**IIa**	In patients with spontaneous type 1 Brugada electrocardiographic pattern and symptomatic VA who either are not candidates for or decline an ICD, quinidine or catheter ablation is recommended.	**I**	We recommend catheter ablation of monomorphic VT in patients with **nonischemic** cardiomyopathy in whom treatment with sotalol or amiodarone has been ineffective.	**Second line**
Patients with CCAD and hemodynamically well-tolerated SMVT and LVEF ≥ 40% as an alternative to ICD therapy.	**IIa**	In patients with verapamil-sensitive, idiopathic left VT related to interfascicular re-entry for whom antiarrhythmic medications are ineffective, not tolerated, or not the patient’s preference, catheter ablation is useful.	**I**		
Patients with CCAD and recurrent and catheter ablation just before (or immediately after) ICD implantation to decrease subsequent VT burden and ICD shocks.	**IIb**	For patients with recurrent episodes of idiopathic VF initiated by PVCs with a consistent QRS morphology, catheter ablation is useful.	**I**		
Patients with DCM/HNDCM with recurrent symptomatic SMVT, or ICD shocks for SMVT in whom AADs are ineffective, contraindicated, or not tolerated.	**IIa**	For patients who require arrhythmia suppression for symptoms or declining ventricular function suspected to be due to frequent PVCs and for whom AADs are ineffective, not tolerated, or not the patient’s preference, catheter ablation is useful.	**I**		
Selected patients with HCM and recurrent, symptomatic SMVT, or ICD shocks for SMVT, in whom AADs are ineffective, contraindicated, or not tolerated.	**IIb**	In patients with adult congenital heart disease with recurrent sustained monomorphic VT or recurrent ICD shocks for VT, catheter ablation can be effective.	**IIa**		
Patients with ARVC and recurrent symptomatic SMVT, or ICD shocks for SMVT despite betablockers.	**IIa**	In patients with bundle-branch re-entrant VT, catheter ablation is useful for reducing the risk of recurrent VT and ICD shocks.	**I**		
Patients with congenital heart disease and recurrent, symptomatic SMVT or ICD shocks for SMVT not manageable by medical therapy or ICD reprogramming.	**IIa**	In patients with SHD who have failed endocardial catheter ablation, epicardial catheter ablation can be useful for reducing the risk of recurrent monomorphic VT.	**IIa**		
Patients with congenital heart disease and repaired TOF, with SMVT or recurrent symptomatic appropriate ICD therapy for SMVT.	**I**				
Patients with congenital heart disease and repaired TOF, with preserved biventricular function and symptomatic SMVT, as alternative to ICD therapy.	**IIb**				
Programmed electrical stimulation with standby catheter ablation in patients with aortic valve disease and SMVT to identify and ablate bundle re-entrant VT, especially if it occurs during a valve intervention.	**I**				
Recurrent episodes of idiopathic VF triggered by a similar PVC non-responsive to medical treatment.	**IIa**				
Catheter ablation of triggering PVCs and/or RVOT substrate in Brugada syndrome patients with recurrent appropriate ICD shocks refractory to drug therapy.	**IIa**				
Symptomatic patients with neuromuscular diseases with bundle re-entrant VT.	**I**				
Post-myocarditis patients with recurrent, symptomatic SMVT or ICD shocks for SMVT, in whom AADs are ineffective, contraindicated, or not tolerated.	**IIa**				
Patients with hemodynamically well-tolerated SMVT occurring in the chronic phase of myocarditis with preserved LV function and a limited scar amenable to ablation, as an alternative to ICD therapy.	**IIb**				
Cardiac sarcoidosis ICD recipients with recurrent, symptomatic SMVT or ICD shocks for SMVT, in whom AADs are ineffective, contraindicated, or not tolerated.	**IIb**				
First-line treatment for symptomatic, idiopathic VT/PVCs from the RVOT.	**I**				
First-line treatment for symptomatic, idiopathic VT/PVCs from the left fascicles.	**I**				
Catheter ablation for symptomatic, idiopathic VT/PVCs from an origin other than RVOT and left fascicles.	**IIa**				
Asymptomatic patients with >20% of idiopathic PVCs per day repeatedly at follow-up.	**IIb**				
Cardiomyopathy suspected to be caused by frequent and predominantly monomorphic PVCs.	**I**				
SHD patients in whom predominantly monomorphic frequent PVCs are suspected to be contributing to the cardiomyopathy.	**IIa**				
Non-responders to CRT with frequent predominantly monomorphic PVCs limiting optimal biventricular pacing despite pharmacological treatment.	**IIa**				

Abbreviations: ESC—European Society of Cardiology; AHA—American Heart Association; ACC—American College of Cardiology; HRS—Heart Rhythm Society; CCS—Canadian Cardiovascular Society; CHRS—Canadian Heart Rhythm Society; LoR—level of recommendation; VT—ventricular tachycardia; AADs—antiarrhythmic drugs; SMVT—sustained monomorphic VT; SPVT—sustained polymorphic VT; VF—ventricular fibrillation; PVC—premature ventricular contraction; ICD—implantable cardioverter defibrillator; DCM—dilated cardiomyopathy; HNDCM—hypokinetic non-dilated cardiomyopathy; HCM—hypertrophic cardiomyopathy; CCAD—chronic coronary artery disease; LVEF—left ventricular ejection fraction; ARVC—arrhythmogenic right ventricular cardiomyopathy; TOF—tetralogy of Fallot; LV—left ventricle; RVOT—right ventricle outflow tract; SHD—structural heart disease; CRT—cardiac resynchronization therapy; VA—ventricular arrhythmias.

## Data Availability

Not applicable.
